# Development and pilot testing of an adaptable protocol to address postpartum depression in pediatric practices serving lower-income and racial/ethnic minority families: contextual considerations

**DOI:** 10.1186/s43058-020-00049-x

**Published:** 2020-07-21

**Authors:** Sarah L. Goff, Michael J. Moran, Kathleen Szegda, Tina Fioroni, Mary Ann DeBanate, Nancy Byatt

**Affiliations:** 1 Department of Health Promotion and Policy, School of Public Health and Health Sciences, University of Massachusetts Amherst, 715 N. Pleasant St., Amherst, MA 01003 USA; 2 Institute for Healthcare Delivery and Population Sciences, University of Massachusetts Medical School-Baystate, 3601 Main St., Springfield, MA 01199 USA; 3 Public Health Institute of Western Massachusetts, 127 State St. 4th Fl., Springfield, MA 01103 USA; 4 Codman Square Health Center, 637 Washington St., Dorchester, MA 02124 USA; 5 Department of Psychiatry, University of Massachusetts Medical School-Worcester, 55 Lake Avenue N, Worcester, MA 01655 USA; 6 Department of Obstetrics and Gynecology, University of Massachusetts Medical School-Worcester, 55 Lake Avenue N, Worcester, MA 01655 USA; 7 Massachusetts Child Psychiatry Access Program for Moms, 25 Staniford Street, Boston, MA 02114 USA

**Keywords:** Postpartum depression, Screening, Pediatric practice, Implementation, Context, Disparities

## Abstract

**Background:**

Postpartum depression (PPD) affects approximately 25% of women in lower-income and racial/ethnic minority populations in the USA. Evidence-based interventions for PPD screening and treatment exist, but many women with PPD are not identified or are inadequately treated. To address this gap, the American Academy of Pediatrics recommends screening for PPD at routine preventive visits in the first 6 months of postpartum, but less than half of pediatricians do so. Small PPD screening studies have been conducted in pediatric practices serving average-risk women, but less is known about practices serving families with lower-income and/or racial/ethnic minority status (safety-net practices). Study objectives were (1) to develop and pilot test an adaptable PPD screening protocol in safety-net practices and (2) to test strategies for implementing the protocol.

**Methods:**

The Consolidated Framework for Implementation Research was used for this two-phase pilot study. Phase I focus groups with pediatric providers and staff in four safety-net practices informed phase II development and implementation of a PPD screening and referral protocol. Feasibility measures included the percentage of eligible women screened and documentation of follow-up plans in the electronic health record at 1-, 2-, 4-, and 6-month preventive visits over 3 months. Implementation strategies were assessed for acceptability, appropriateness, and feasibility.

**Results:**

Focus group participants felt that (1) addressing PPD in the pediatric setting is important, (2) all clinical team members should be engaged in screening, (3) workflows and competing interests may present barriers, and (4) commonly used screening tools/approaches may not adequately detect depression in the population studied. During protocol implementation, screening rates increased from 75 to 85% for 324 eligible preventive visits and documentation of follow-up plans increased from 66 to 87%. Only 6.5% of women screened positive (EPDS ≥ 10). Minor adaptations to implementation strategies were recommended to improve acceptability, appropriateness, and feasibility.

**Conclusions:**

Although developing and implementing an adaptable protocol for PPD screening in safety-net pediatric practices using external facilitation and a bundle of implementation strategies appear feasible, low positive screen rates suggest adaptations to account for intersecting patient, practice, and external policy contexts are needed to improve PPD screening effectiveness in these practices.

Contributions to the literature

- Pediatric healthcare providers in practices serving families with lower-income and racial/ethnic minority status in the USA agreed that screening mothers for postpartum depression (PPD) at preventive health visits for 1- to 6-month-old infants is important, but also had concerns that commonly used screening instruments and processes may fail to identify women with PPD in their practice settings.
- Facilitated development and implementation of a PPD screening and referral protocol tailored to a pediatric practice’s needs may overcome some barriers to PPD screening in pediatric practices serving families with lower-income and racial/ethnic minority status.
- The lower-than-expected percentage of women with positive screens for PPD found in this study (6.5% vs 25%) suggests that efforts to improve PPD screening in pediatric practices serving families with lower-income and racial/ethnic minority status may require adaptations to both the screening tool and strategies to implement it to account for contextual factors.

## Introduction

One in seven women overall and as many as one in four women in lower-income and racial/ethnic minority populations in the USA develop postpartum depression (PPD), also known as postnatal depression, in the year following the birth of a child [1–4]. While other factors, such as prior depression or being a mother of multiples, also increase risk for PPD, this study focuses on women with lower-income and racial/ethnic minority status because of the health disparities experienced by these populations in the USA. The US Preventive Services Task Force, the American College of Obstetricians and Gynecologists, and the American Academy of Pediatrics recommend universal screening for PPD across maternal and child healthcare settings [5–7]. Evidence-based practices for PPD screening and treatment exist [8–11], but the vast majority of women go undiagnosed or are inadequately treated [12]. The consequences of untreated PPD include impaired relationships, diminished social function, higher rates of suicide and infanticide, and lost work days [13]. PPD also has long-lasting negative impacts on child development [14]. Barriers to effective screening and referral for treatment have been described at the patient (e.g., stigma, trauma, language, literacy) [15], provider (e.g., inadequate training) [7], and system (e.g., work-flow, mental health referrals, insurance) levels [10, 16, 17]. Some barriers may have a greater impact on populations that experience health disparities.

Infants are seen by a pediatric provider (physician or advance practitioner (AP)) for routine preventive care seven or more times in the first 12 months of life, presenting numerous opportunities for PPD screening [18]. However, fewer than half of pediatric providers routinely screen for PPD [7]. Strategies to improve PPD screening and referral in the pediatric setting have been tested in small US-based quality improvement studies [19–22], but the studies have taken place primarily in practices serving average-risk populations and have focused on changes in screening rates alone. This study’s overall aim was to develop and pilot test an intervention to improve PPD screening and referral in pediatric practices that serve predominantly lower-income and racial/ethnic minority families (safety-net practices—referred to as “practices” throughout the manuscript). This manuscript focuses on the screening aspects of the study. Referral will be reported elsewhere so that the complexities of identifying and addressing challenges to implementing both screening and referral can be fully described and the implications for research and clinical care can be fully explicated for each as well.

## Methods

### Overview

**Fig. 1:**
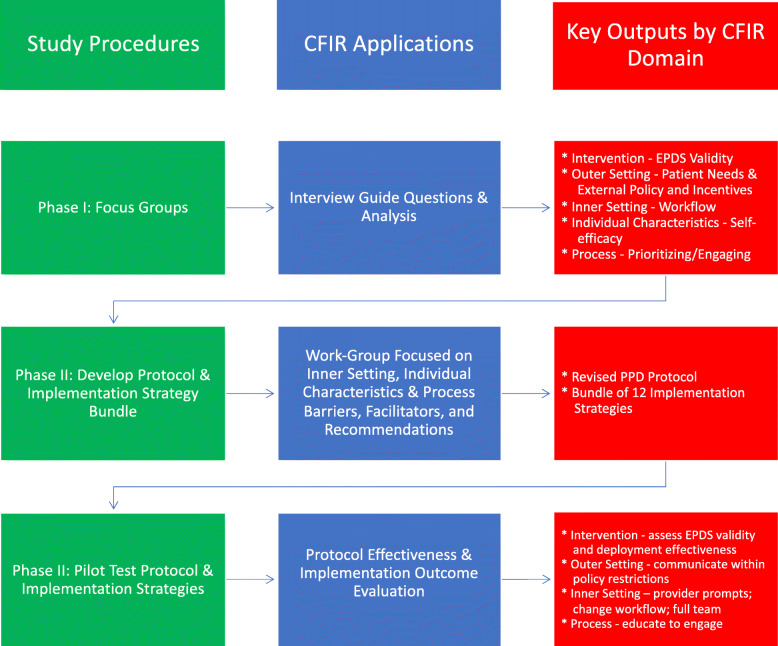
Integration of CFIR across stages of the study

The objectives of this two-phase study were (1) to develop and pilot test an adaptable PPD screening protocol for safety-net practices and (2) to pilot test a bundle of strategies for implementing the protocol. In phase I, formative focus groups were conducted at four safety-net practices. In phase II, focus group data were used to develop a protocol for PPD screening and pilot test the protocol and a bundle of strategies to implement the protocol. The study was approved by the University of Massachusetts Medical School-Baystate Institutional Review Board. The Consolidated Framework for Implementation Research (CFIR) [23] served as the conceptual and analytic framework for this mixed-methods pilot study. CFIR was selected because its five domains (intervention characteristics, outer setting, inner setting, characteristics of individuals, and process of implementation) are pertinent to the implementation of complex interventions such as screening for PPD in safety-net practices, and its flexible structure is designed to be used across all phases of the study (Fig. 1). An advisory board was convened for the study so that the perspectives of stakeholders outside of the healthcare system could be included. The board included the director for research and evaluation of a community-based public health organization, a behavioral health administrator, and the leader of a county-wide perinatal depression coalition. The Board met with the lead investigators (SG and NB) via telephone twice monthly to provide input on design, review study materials, and help interpret data.

### Phase I: Focus groups

#### Sampling and recruitment

Safety-net practices (referred to as “practices” throughout the manuscript) located in the county in which the study was conducted were purposively sampled to include practice characteristics that could potentially facilitate or hinder efforts to implement a PPD screening protocol, such as practice size, presence of a co-located behavioral health team, and organizational structure (e.g., large integrated health care system, managed care organization). An e-mail was sent to practice leaders inviting their practice to participate; the study was then explained further by phone and a time for the focus group scheduled if indicated.

#### Focus group procedures and analysis

Focus groups were conducted using a semi-structured interview guide that was pre-tested with the Advisory Board. Interview questions explored topics related to all five CFIR domains (Fig. 1) [15, 23]. Interview questions aimed to understand current approaches to PPD screening and referral, identify perceived barriers and facilitators to effective PPD screening and referral, and elicit recommendations for best approaches to developing and implementing PPD protocols in this setting. Focus groups took place at practice sites, lasted approximately 1 h, and were facilitated by the study’s lead investigator, a physician-investigator experienced in using qualitative methods. A trained research assistant (MM) took field notes [24], and the sessions were audiotaped and professionally transcribed.

Data were analyzed using qualitative content analysis, a method commonly used when there is existing knowledge about a phenomenon [25]. An a priori codebook was created using the five CFIR domains [23]. SG and MM applied codes (labels to categorize small portions of the text) to transcripts iteratively, adding new codes when indicated. Codes were then sorted into major themes (groups of codes that describe a broader phenomenon) and sub-themes; the analysis was supported by the Dedoose analytic software [26]. Data saturation was achieved as evidenced by no new themes emerging in the final two focus groups. Major themes were compared to prior studies of PPD screening in the pediatric setting to ascertain support for concepts previously identified and to identify new concepts.

### Phase II: Protocol development and pilot testing of protocol and implementation strategies

One of the phase I practices was selected to serve as the phase II test site based on its organizational complexity (urban pediatric residency program), readiness for implementation based on its engagement in phase I, and interest in developing and implementing a PPD protocol. The test site had 11 general pediatric providers and 27 pediatric residents; more than 90% of its patients were insured by Medicaid (state-sponsored insurance for lower-income families); the majority of patients were African-American or Hispanic and spoke a primary language other than English. Potential facilitators and barriers to implementing evidence-based PPD screening identified within CFIR domains in phase 1 informed protocol development and implementation (Fig. 1).

Twelve implementation strategies, drawn from the Expert Recommendations on Implementing Change [27], were tested as a bundle in the study. The strategies, listed in Table 1 and numbered in parentheses in the text, were selected through an iterative process that included the investigators, the Advisory Board, and the multi-disciplinary PPD workgroup convened at the test site for the study (#1). The workgroup included a pediatric provider “champion” (#2), a front desk staff member, a nurse, a behavioral health clinician, and a community health worker. The group met twice monthly for the duration of the study, and meetings were facilitated (#3) by members of the investigative team.

**Table 1:** Implemantation strategies included in the strategy bundle bundle

Implementation strategies	
1. Assess for readiness and identify barriers and facilitators	
2. Audit and provide feedback	
3. Conduct educational meetings	
4. Conduct local consensus discussions	
5. Conduct local needs assessment	
6. Facilitation	
7. Identify and prepare champions	
8. Use advisory boards and workgroups	
9. Tailor strategies	
10. Remind clinicians	
11. Involve patients/consumers and family members	
12. Use train-the-trainer strategies	

#### Screening protocol development

The workgroup members first decided on the desired outcomes for the newly implemented PPD screening protocol. During this process, they discovered that a PPD protocol had been developed in the practice several years previously but was not being used. The prior protocol included guidelines for when to screen mothers and recommended actions based on screening results. The workgroup felt the prior protocol had too little information, was difficult to interpret, had outdated information, and had not been implemented well. They decided to revise the existing protocol, taking barriers and recommendations from phase I focus groups into account and addressing additional potential barriers identified during workgroup planning meetings using CFIR as a guide (Fig. 1). The workgroup developed and delivered educational and informational sessions to the pediatric providers, residents, and staff in the practice that included efforts to address barriers. These sessions also aimed to gather input, identify additional perceived barriers and facilitators, and build consensus on the need for and approach to revising and implementing the PPD protocol (#4, #5, #6, #7). In addition, the practice’s Patient and Family Advisory Council was consulted to obtain patient perspectives (#8). Prior to the study, the practice screened mothers for PPD using the Edinburgh Postnatal Depression Scale (EPDS) [28] as part of a developmental screening packet and continued using this instrument during the study. Sensitivity and specificity of the 10-item EPDS vary depending on the population screened; however, a cutoff score of 10 (out of 30) is generally considered sufficiently sensitive and specific to indicate a high likelihood of PPD [29].

#### Development and Pilot-testing of Implementation Strategies

In addition to the strategies used during protocol development, the workgroup tailored their selection of implementation strategies to their practice’s needs (#9) and included texting providers bi-weekly with individual reports on screening performance (#10), creating a PPD toolkit that included the protocol and community resources and placing it in each exam room as a reminder for providers (#10), and providing mothers a brief written rationale for screening on the cover of the screening tool (#11). The facilitators provided additional training for workgroup members to support them in training others in the practice to use the protocol (#12).

#### Measures

Measures to assess uptake of the protocol included a review of the practice’s electronic health record (EHR) to determine whether a completed EPDS form (screening tool) was present, the pediatric provider documented that the screening results had been reviewed, and a follow-up plan was documented for women with positive screens. These data were extracted for all preventive care visits for 1-, 2-, 4-, and 6-month-old infants that occurred at the test site between May 1 and June 30, 2018 (pre-implementation), and October 1 through December 31, 2018 (implementation). The percentage of EPDS scores that were 10 or higher (high likelihood of PPD) was also calculated. Measures to evaluate the implementation strategy bundle included acceptability, appropriateness, and feasibility [30] and were assessed: by eliciting and documenting real-time feedback on the implementation strategies and process from providers and staff verbally during standing practice meetings and via e-mail, and by observations made by the facilitators and workgroup over the course of the study.

## Results

### Phase I: Focus groups

Focus groups were conducted with a total of 28 pediatric providers and staff at four of the five practices invited to participate. The practices ranged in size from four to 38 providers (including residents); three were part of a large integrated health system and one was a multi-site, multi-specialty practice. Additional practice and provider data are located in Table 2.

**Table 2:** Focus group participant demographics

Participant characteristics (*N* = 28)	*N* (%)
Age, mean (range)	46 (28–64)
Female	24 (86)
Self-identified race/ethnicity	
Caucasian	19 (68)
Asian	3 (11)
African-American	2 (7)
Hispanic	2 (7)
Multi-racial	2 (7)
Professional title/position	
Physician	13 (46)
Nurse practitioner	4 (14)
Community health worker	2 (7)
Certified nurse-midwife	2 (7)
Resident physician	2 (7)
Behavioral health clinician	1 (4)
Medical assistant	1 (4)
Physician’s assistant	1 (4)
Registered nurse	1 (4)
Schedule coordinator	1 (4)
Years in profession/position, mean (range)	14 (1 month–35 years)
Years at practice, mean (range)	9 (1 month–31 years)

Potential facilitators and barriers to implementing evidence-based PPD screening in practices serving families with lower-income and racial/ethnic minority status were identified in the following CFIR domains: (1) intervention characteristics, (2) inner setting, (3) outer setting, and (4) processes. Linkages between these findings and protocol development and implementation are depicted in Fig. 1. Selected sub-themes for these domains are described below with illustrative quotes; additional sub-themes and quotes are located in Table 3.

**Table 3:** Major themes and sub-themes with illustrative quotes

CFIR domain and sub-theme	Quotes
1. Intervention	
Appropriateness of pediatric providers screening for PPD	**“In the pediatric world, you get your patient, you get their family, you get the constellation.”** “The health of the mother affects the health of the child.” “For pediatrician as that piece…we have like twice the number of opportunities to screen.”
Perception of mothers’ reactions to screening	**“I think if you have a rapport with a family, it is so much better…I do think people open up more readily if I know the mom and the other children… you can even sense it more than someone you don’t know.”** “Sometimes they’re a little confused because they don’t expect us to ask that, because they think that we’re here to provide care for the baby.” “I think by the sixth-month visit, sometimes they’ve gone, why are you asking me?”
Screening tools	“… are there different questions we should be asking them based on cultural identify that might be more specific to them in how they are experiencing postpartum depression… just wondering how many we are missing based on the way we are asking the questions…” “You are asking her a whole bunch of questions. You can see that she is exhausted, you can see that she is stressed, and you do reflective questioning…You get a sense, outside of those questions, then, when you are dealing with them on a human level and you are really responding to where they are at, they are more likely to say, ‘Yes, I would talk to somebody about that. Yes, I could need some help with that’.”
2. Inner setting	
Practice size	“It is just hard to do in our setting. I do think people open up more readily if I know the mom and the other children.”
Electronic medical record (EMR)	“If they’re in our system, we can easily send a letter, you know, a quick note over to the OB and say can you please get this one in, but when they’re not part of [our system] is when it becomes a nightmare.” “… if we expanded pediatrics to perinatal and OB into the baby… We[could] sign a release, the mom signs off a release, and offices talk. When they score on a peri-birth and it is positive, that goes in the baby’s chart.”
Team responsibility	“We are still in a silo mode of ‘this is OB and this is pediatrics’. I think we are crossing into that idea that we are going to get more involved with the women’s clinic... and more involved in the prenatal care…”
Internal linkages to behavioral health	“They [behavioral health clinicians in the system] pretty much only see private patients, so…we rarely use our own mental [health services]…”
3. Outer setting	
Behavioral health resources	“It’s helpful [to have co-located behavioral health care], but so I just wonder, how the other end of [the service] works, I don’t know.” “I think we would feel better if we could call [the service] ourselves…[We] get the sense that nothing’s going to really happen. You already have a therapist, but apparently…it has to be their provider who calls. They’ve made that quite clear.” “They just send us this list of some names and phone numbers, but sometimes even with that list, our patients have a lot of trouble and people don’t return their calls.” [the pediatrician] “…can’t make the referral because the mother’s not [their] patient.”
Privacy laws	[pediatric providers ]“… have no idea at all what is going on…[with a mother’s healthcare across the health system]… [information relating to mothers is] an important piece [of caring for their children]”
4. Processes	
Positive screens	“I am worried if I get a positive [screen], what to do next.” “[With behavioral health services] you get advice…but you don’t always know when somebody’s going where. And you don’t get, you often – you may get that immediate [response], but after that it’s still a black hole.”
Preventive care visit priorities	“I have to remember [to screen] and if the visit gets complicated it may not be something that crosses my mind until they have left the room and I realize we didn’t check it.” “Sometimes it [depression symptoms] comes up at the very end [of a visit] and we’re behind and it becomes challenging to address.”

#### Intervention characteristics—screening tools

Although participants felt that it was important for pediatric practices to screen mothers for PPD, many also felt that commonly used screening instruments such as the EPDS [28] might not accurately identify women at risk for PPD in lower-income and racial and ethnic minority populations. Some felt that cultural factors and low literacy levels might make screening instruments hard for some women to complete, “I don’t know if the depression questions often resonate … like if there’s cultural differences with how they’re interpreted or it’s a literacy thing.” Several participants also thought that mothers might be afraid that disclosing depressive symptoms would risk drawing the attention of child protection services. Many felt that verbal screening might be more effective and that having a prior positive connection with a mother likely increased comfort with the disclosure of depressive symptoms.

#### Inner setting—a team responsibility

Participants almost unanimously felt that screening would be most effective if the entire clinical team was involved, “I think it is everyone [on the clinical team who is responsible for screening]. It starts from the get-go when they come in, through the MAs [medical assistants] and then, if you notice something, you relay that on to the provider.” Some participants felt that the front desk staff were more likely than the pediatric provider to see that a mother is stressed or having difficulty and may have more insight into her life situation because they are from the same community. They also felt that the front desk staff may more easily gain the trust of patients.

#### Outer setting—privacy laws

Many participants felt that privacy laws presented barriers to identifying women at risk for PPD because the laws inhibit the exchange of patient information between pediatricians and women’s healthcare providers, “I think probably one of the biggest barriers is HIPAA and crossing the barrier between ‘Mom is not my patient’ which… from a provider standpoint is stupid because the mom and the baby are linked…so, the fact that something… is [affecting] the baby because of mom [but I can’t] reach out to mom doesn’t make a lot of sense.”

#### Processes—preventive care visit priorities

Several participants commented that they felt that PPD screening was not currently a high priority during preventive visits and that time pressures presented barriers to conducting adequate PPD screening, especially during preventive visits for families with complex social needs, “It is just a matter of saying, ‘This is a priority and put it through the system’, but we haven’t at this point.”

### Phase II: Protocol development and pilot testing of protocol and implementation strategies

At baseline, completed EPDS forms were present in the EHR for 75% (58/77) of eligible visits, and documentation of EPDS review was present for 66% (38/58) of those with completed screens. Following the implementation of the new protocol, PPD screening was completed at 86% of the 324 eligible well-child visits that occurred between October 1 and December 31, 2018. Of these, 86% (238/278) had documentation that the EPDS responses had been reviewed. Only 6.5% (18/278) of the completed screens had a score of 10 or greater (high likelihood of PPD). Qualitative feedback from pediatric providers and the Patient and Family Advisory Council suggested that low rates might be accounted for by cultural differences on how depression is perceived and potentially stigmatized and mothers’ concerns about drawing the attention of child protective services.

The majority of providers and staff who gave feedback felt that the bundle of implementation strategies used was appropriate, but some felt that more education about PPD and how to use the EPDS would be helpful. One physician felt that neither the new protocol nor the strategies were appropriate because she believed that the current screening processes were adequate and that the workgroup should focus on being sure providers bill for the screening. Assessment of acceptability of the implementation strategies showed that most providers felt that the feedback on screening was effective, but preferred having the feedback sent by text rather than by e-mail. Some also felt that the practice’s standard communication processes slowed the diffusion of information about the new protocol and its implementation. Although pediatric providers and staff reported that the process of revising and implementing the PPD protocol was feasible with the support of the investigative team, they expressed concerns that the process might not be feasible without the external facilitation.

## Discussion

The failure to systematically implement effective PPD screening in safety-net pediatric practices in the USA means that women from lower-income and/or racial and ethnic minority groups are more likely to develop PPD and to experience the deleterious effects of untreated PPD for both themselves and their children. Racial and ethnic disparities in maternal mortality rates in the USA [31] and the association of maternal mental health with these high mortality rates make delivering evidence-based PPD care to higher-risk women even more of a priority. Facilitated development and implementation of a PPD screening protocol in a safety-net practice proved feasible in this study as evidenced by the increase in the percentage of women screened and documentation of plans following screening. The bundle of implementation strategies tested was largely found to be applicable, acceptable, and feasible with some adaptations. However, the unanticipated finding of a much lower-than-expected percentage of women with positive screens raised important new questions about the validity of a commonly used PPD screening tool and how best to deploy the tool in pediatric practices serving lower-income and racial and ethnic minority families.

The EPDS is one of the most commonly used PPD screening tools in primary care settings and was found to have a sensitivity of 86%, specificity of 78%, and positive predictive value of 73% when it was first developed [28]. Subsequent studies of its psychometric properties have found that the EPDS may perform differently in some patient populations [32–35]. The investigators who developed the EPDS have cautioned that its psychometric properties should be evaluated for different testing conditions [36], but this is time-consuming, costly, and often not done. The lower-than-expected percentage of women with positive screens for depressive symptoms found in the current study could potentially be explained by the concerns expressed during the focus groups that some women may be less comfortable disclosing depressive symptoms to their child’s pediatric provider than they would be with their own provider if those concerns are validated. Focus group participants further hypothesized that if some women do in fact feel uncomfortable, the discomfort might stem from a lack of trust in the healthcare system, cultural and linguistic factors, and fear of involvement of child protection service, consistent with theoretical concerns expressed in prior studies [3, 37, 38]. These findings suggest that increasing the percentage of women screened for PPD in safety-net pediatric practices may be necessary, but not sufficient to address disparities in PPD care.

In addition to finding low positive screen rates, this study identified contextual factors in several CFIR domains and at multiple levels that may need to be considered when implementing interventions to improve PPD care for women in the populations and practice setting studied. Some of the factors identified, such as difficulties coordinating care when a patient is not the referring provider’s patient and the effects of stigma related to mental health disorders, concur with prior studies [10, 17, 32]. The current study also identified barriers created by privacy regulations, which pediatric providers felt limited their capacity to advocate for women who may have greater challenges navigating the mental healthcare system due to language, literacy, transportation, and insurance status barriers. Health policies and practice structures that address these and other barriers, such as EHR prompts that require PPD screening results to be entered before moving to other parts of the EHR, may help improve the translation of evidence-based PPD care into practice in this setting.

Limitations of the study include recruitment from one geographic location since studies in other practices and other regions might find different contextual barriers to screening. However, the study was designed to create a flexible process for protocol development and implementation that could be adapted in different settings. Conducting focus groups with postpartum women was outside of the scope of the study, but this would likely help to further understand contextual barriers to PPD screening beyond what we learned from members of the Patient and Family Advisory Council. We collected qualitative data for implementation outcomes for this pilot study, but a systematic mixed-methods assessment of these outcomes would be of use when scaling up this study. This study was not designed to test the effectiveness of individual implementation strategies, but future studies may compare the effectiveness of different bundles of strategies. Sustainment of changes beyond the pilot study period was outside of the scope of the study but will be an important implementation outcome to measure in future studies. Finally, interventions to improve screening for other high-risk groups of women (e.g., a prior diagnosis of depression or mothers of multiples, or preterm infants) may require different considerations than those identified in this study.

## Conclusions

This study demonstrated the feasibility of developing and implementing an adaptable protocol for PPD screening in pediatric practices serving families with lower-income and/or racial and ethnic minority status using external facilitation and a bundle of implementation strategies selected by a practice site. However, it also raised some important questions about how contextual factors at the intersection of inner setting (e.g., pediatric (vs adult) practice, workflows) and outer setting (e.g., low-income and racial/ethnic minority population needs, child protection service policies) may reduce the sensitivity of PPD screening instruments and may require adaptations to how screening instruments are deployed. Although further study will be needed to better understand the effect of these contextual factors, this study suggests that in-person screening by a trusted person rather than completion of a paper form before seeing the pediatric provider may increase the sensitivity of the EPDS. The study also suggests that PPD screening may be enhanced by systems-level changes in practice structures, such as co-locating obstetric and pediatric care, adapting workflows to prioritize PPD screening, and engaging all pediatric healthcare team members in PPD care.

## Data Availability

The data generated during this study are available from the corresponding author upon reasonable request.

## Abbreviations

CFIR: Consolidated Framework for Implementation Research
EHR: Electronic health record
EPDS: Edinburgh Postnatal Depression Scale
PPD: Postpartum depression

## References

[1] Howell EA, Mora PA, Horowitz CR, Leventhal H (2005). Racial and ethnic differences in factors associated with early postpartum depressive symptoms. Obstet Gynecol.

[2] Yim IS, Tanner Stapleton LR, Guardino CM, Hahn-Holbrook J, Dunkel SC (2015). Biological and psychosocial predictors of postpartum depression: systematic review and call for integration. Annu Rev Clin Psychol.

[3] Kozhimannil KB, Trinacty CM, Busch AB, Huskamp HA, Adams AS (2011). Racial and ethnic disparities in postpartum depression care among low-income women. Psychiatr Serv Wash DC.

[4] O’Hara MW, McCabe JE (2013). Postpartum depression: current status and future directions. Annu Rev Clin Psychol.

[5] Siu AL (2016). US Preventive Services Task Force (USPSTF), Bibbins-Domingo K, Grossman DC, Baumann LC, Davidson KW, et al. Screening for depression in adults: US Preventive Services Task Force Recommendation Statement. JAMA..

[6] Screening for Perinatal Depression - ACOG [Internet]. 2016 [cited 2016 May 30]. Available from: http://www.acog.org/Resources-And-Publications/Committee-Opinions/Committee-on-Obstetric-Practice/Screening-for-Perinatal-Depression.

[7] Earls MF (2010). Committee on Psychosocial Aspects of Child and Family Health American Academy of Pediatrics. Incorporating recognition and management of perinatal and postpartum depression into pediatric practice. Pediatrics..

[8] Bobo WV, Yawn BP (2014). Concise review for physicians and other clinicians: postpartum depression. Mayo Clin Proc.

[9] Fitelson E, Kim S, Baker AS, Leight K (2010). Treatment of postpartum depression: clinical, psychological and pharmacological options. Int J Women's Health.

[10] Gjerdingen DK, Yawn BP (2007). Postpartum depression screening: importance, methods, barriers, and recommendations for practice. J Am Board Fam Med JABFM.

[11] O’Connor E, Rossom RC, Henninger M, Groom HC, Burda BU (2016). Primary care screening for and treatment of depression in pregnant and postpartum women: evidence report and systematic review for the US Preventive Services Task Force. JAMA..

[12] Byatt N, Xiao RS, Dinh KH, Waring ME (2016). Mental health care use in relation to depressive symptoms among pregnant women in the USA. Arch Womens Ment Health.

[13] Putnam KT, Wilcox M, Robertson-Blackmore E, Sharkey K, Bergink V, Munk-Olsen T (2017). Clinical phenotypes of perinatal depression and time of symptom onset: analysis of data from an international consortium. Lancet Psychiatry.

[14] Letourneau NL, Dennis C-L, Cosic N, Linder J (2017). The effect of perinatal depression treatment for mothers on parenting and child development: a systematic review. Depress Anxiety.

[15] Byatt N, Biebel K, Friedman L, Debordes-Jackson G, Ziedonis D (2013). Women’s perspectives on postpartum depression screening in pediatric settings: a preliminary study. Arch Womens Ment Health.

[16] Accortt EE, Wong MS (2017). It is time for routine screening for perinatal mood and anxiety disorders in obstetrics and gynecology settings. Obstet Gynecol Surv.

[17] Byatt N, Levin LL, Ziedonis D, Moore Simas TA, Allison J (2015). Enhancing participation in depression care in outpatient perinatal care settings: a systematic review. Obstet Gynecol.

[18] Committee on Practice and Ambulatory Medicine, Bright Futures Periodicity Schedule Workgroup. 2017 Recommendations for Preventive Pediatric Health Care. Pediatrics. 2017 Apr;139(4). 10.1542/peds.2015-2009 26324870

[19] Olin S-CS, McCord M, Stein REK, Kerker BD, Weiss D, Hoagwood KE, et al. Beyond screening: a stepped care pathway for managing postpartum depression in pediatric settings. J Women's Health 2002. 2017;26(9):966–975. 10.1089/jwh.2016.6089 PMC5749581 28409703

[20] Mgonja S, Schoening A (2017). Postpartum depression screening at well-child appointments: a quality improvement project. J Pediatr Health Care Off Publ Natl Assoc Pediatr Nurse Assoc Pract.

[21] Puryear LJ, Nong YH, Correa NP, Cox K, Greeley CS. Outcomes of implementing routine screening and referrals for perinatal mood disorders in an integrated multi-site pediatric and obstetric setting. Matern Child Health J. 2019. 10.1007/s10995-019-02780-x 31222600

[22] Russomagno S, Waldrop J (2019). Improving postpartum depression screening and referral in pediatric primary care. J Pediatr Health Care.

[23] Damschroder LJ, Aron DC, Keith RE, Kirsh SR, Alexander JA, Lowery JC (2009). Fostering implementation of health services research findings into practice: a consolidated framework for advancing implementation science. Implement Sci IS.

[24] Corbin JM, Strauss, Strauss AL. Basics of qualitative research: techniques and procedures for developing grounded theory. Los Angeles, Calif.: Sage Publications; 2008.

[25] Hsieh H-F, Shannon SE (2005). Three approaches to qualitative content analysis. Qual Health Res.

[26] Dedoose Version 8.0.35, web application for managing, analyzing, and presenting qualitative and mixed method research data (2018). Los Angeles, CA: SocioCultural Research Consultants, LLC www.dedoose.com. Dedoose.

[27] Powell BJ, Waltz TJ, Chinman MJ, Damschroder LJ, Smith JL, Matthieu MM (2015). A refined compilation of implementation strategies: results from the Expert Recommendations for Implementing Change (ERIC) project. Implement Sci IS.

[28] Cox JL, Holden JM, Sagovsky R (1987). Detection of postnatal depression. Development of the 10-item Edinburgh Postnatal Depression Scale. Br J Psychiatry J Ment Sci.

[29] Eberhard-Gran M, Eskild A, Tambs K, Opjordsmoen S, Samuelsen SO (2001). Review of validation studies of the Edinburgh Postnatal Depression Scale. Acta Psychiatr Scand.

[30] Proctor E, Silmere H, Raghavan R, Hovmand P, Aarons G, Bunger A (2011). Outcomes for implementation research: conceptual distinctions, measurement challenges, and research agenda. Admin Pol Ment Health.

[31] Pregnancy Mortality Surveillance System | Maternal and Infant Health | CDC [Internet]. 2019 [cited 2019 Dec 17]. Available from: https://www.cdc.gov/reproductivehealth/maternal-mortality/pregnancy-mortality-surveillance-system.htm.

[32] Olin S-CS, Kerker B, Stein REK, Weiss D, Whitmyre ED, Hoagwood K, et al. Can postpartum depression be managed in pediatric primary care? J Womens Health 2002. 2016 Apr;25(4):381–90. 10.1089/jwh.2015.5438 PMC4834523 26579952

[33] Chaudron LH, Szilagyi PG, Tang W, Anson E, Talbot NL, Wadkins HIM (2010). Accuracy of depression screening tools for identifying postpartum depression among urban mothers. Pediatrics..

[34] Matthey S, Agostini F (2017). Using the Edinburgh Postnatal Depression Scale for women and men-some cautionary thoughts. Arch Womens Ment Health.

[35] Gibson J, McKenzie-McHarg K, Shakespeare J, Price J, Gray R (2009). A systematic review of studies validating the Edinburgh Postnatal Depression Scale in antepartum and postpartum women. Acta Psychiatr Scand.

[36] Cox J. Use and misuse of the Edinburgh Postnatal Depression Scale (EPDS): a ten point ‘survival analysis.’ Arch Womens Ment Health 2017;20(6):789–790. 10.1007/s00737-017-0789-7 29101480

[37] Copeland VC, Snyder K (2011). Barriers to mental health treatment services for low-income African American women whose children receive behavioral health services: an ethnographic investigation. Soc Work Public Health.

[38] Edge D (2011). “It’s leaflet, leaflet, leaflet then, ‘see you later’”: black Caribbean women’s perceptions of perinatal mental health care. Br J Gen Pract J R Coll Gen Pract.

